# RIPK3: A New Player in Renal Fibrosis

**DOI:** 10.3389/fcell.2020.00502

**Published:** 2020-06-16

**Authors:** Ying Shi, Xinming Chen, Chunling Huang, Carol Pollock

**Affiliations:** ^1^Department of Nephrology, School of Medicine, Stanford University, Palo Alto, CA, United States; ^2^Kolling Institute of Medical Research, Sydney Medical School, The University of Sydney, Sydney, NSW, Australia

**Keywords:** RIPK3, Receptor interacting serine/threonine-protein kinase 3, renal fibrosis, TGF – β1, necroptosis, dabrafenib

## Abstract

Chronic kidney disease (CKD) is the end result of a plethora of renal insults, including repeated episodes of acute or toxic kidney injury, glomerular, or diabetic kidney disease. It affects a large number of the population worldwide, resulting in significant personal morbidity and mortality and economic cost to the community. Hence it is appropriate to focus on treatment strategies that interrupt the development of kidney fibrosis, the end result of all forms of CKD, in addition to upstream factors that may be specific to certain diseases. However, the current clinical approach to prevent or manage renal fibrosis remains unsatisfactory. The rising importance of receptor-interacting serine/threonine-protein kinase (RIPK) 3 in the inflammatory response and TGF-β1 signaling is increasingly recognized. We discuss here the biological functions of RIPK3 and its role in the development of renal fibrosis.

## Introduction

Chronic kidney disease (CKD) is defined as a loss of glomerular filtration and or proteinuria, persisting for at least 3 months or structural abnormalities in the kidney. In the majority of cases, CKD eventually leads to end-stage kidney disease (ESKD) requiring renal replacement therapy or death will ensue. CKD affects a large proportion of the population and considerably more than is widely appreciated by the general public. In 2016–2017, 1.8 million hospitalizations in Australia were associated with CKD, which accounts for 16% of all hospital admissions in Australia. Of those hospitalizations, 80% were for regular dialysis (AIHW, 2019). Having CKD increases the length of stay, cost, and complications of non-CKD related hospital admissions. In the United States, the overall prevalence of CKD in the general adult population was 14.8% in 2011–2014 (United States Renal Data System, 2018). Regardless of the cause of the initial renal injury, progressive renal fibrosis is common to all forms of CKD, characterized pathologically by extracellular matrix (ECM) accumulation, myofibroblast activation, and inflammatory cell infiltration (Lee and Kalluri, 2010; Carew et al., 2012).

To date, inhibition of the renin-angiotensin-aldosterone system (RAAS) is the crucial strategy utilized to slow deterioration of renal functional decline. However, this influences intrarenal and extrarenal hemodynamics, and only secondarily reduces the development of renal fibrosis. It is primarily beneficial in patients with proteinuric renal disease, and at best, it delays the time to ESKD, leading to renal replacement therapy or death, by a factor of months. More recently, sodium-glucose linked transport inhibitors have been shown to reduce the development of end-stage kidney disease in patients with diabetic kidney disease (Ingelfinger and Rosen, 2019), and a recent trial in both diabetic and non-diabetic CKD was prematurely terminated in light of positive results in favor of the SGLT2 inhibitor (ClinicalTrials.gov, 2020). However, a treatment gap remains, and novel therapies directed toward reducing the ultimate fibrotic response in the kidney are urgently needed to arrest the progression of CKD and improve the outcome of patients.

## Transforming Growth Factor BETA-1 (TGF-β1)

TGF-β is the prototype of a family of secreted polypeptide growth factors. Three isoforms of TGF-β have been identified in mammals, including TGF-β1, TGF-β2 and TGF-β3 (Yu et al., 2003). All TGF-βs are synthesized as homodimeric proproteins together with the latency-associated peptide (LAP), which binds to the TGF-β homodimer to promote the formation of the latent TGF-β binding protein (LTBP) (Robertson et al., 2015). The synthesized complex, consisting of TGF-β dimer, LAP dimer, and LTBP, remains inactive and stored in the ECM (Hinz, 2015). LTBP serves as a localizer to interact with the ECM (Annes et al., 2003). LAP inhibits TGF-β activity by preventing TGF-β binding to its receptors (Annes et al., 2003; Hinz, 2015). This mechanism controls free and, therefore, active TGFβ tissue levels. To cleave the TGF-β complex into the active component and release active TGF-β, one or more of a wide range of proteases, including plasmin, matrix metalloproteinase (MMP) 2, and MMP9, thrombospondin, integrins, and the cationic independent mannose 6 phosphate receptor, are needed (Annes et al., 2003).

It is well accepted that overexpression of active TGF-β1 induces a fibrotic response in multiple organs, including the kidney (Sanderson et al., 1995). TGF-β1 is a well-characterized key mediator in the pathogenesis of tubulointerstitial fibrosis, due to its direct and indirect effect on various cells types (Roberts, 1998; Wang et al., 2005; Bottinger, 2007). The direct action of TGF-β1 includes the transition of cells to a fibroblastic phenotype and synthesis of profibrotic proteins, such as collagens and fibronectin (Border et al., 1990; Haberstroh et al., 1993; Wilson et al., 1993). TGF-β1 also facilitates an indirect fibrotic response, via accelerating apoptosis of resident healthy cells and promoting resident and infiltrating cells to increase ECM deposition (Lebrin et al., 2005; Das et al., 2014; Mack and Yanagita, 2015). Inhibiting TGF-β1 in animal models of kidney disease attenuates fibroblast activation and ECM accumulation (Moon et al., 2006; Russo et al., 2007; Murphy et al., 2012; McGaraughty et al., 2017).

The central signal transduction in response to TGF-β1 is mediated by two specific receptors, TGF-β type II receptor (TGFβRII) and the TGF-β type I receptor (TGFβRI). TGF-β1 firstly binds with TGFβRII in an active form (homodimers) and recruits the low-affinity receptor (TGFβRI) by the ligand-bound high-affinity receptor (TGFβRII) (Groppe et al., 2008). The activation of TGFβRI initiates receptor signaling (Xu et al., 2012a) and phosphorylates the substrates, the Smad proteins. Specifically, TGF-β1 signaling stimulates receptor-regulated Smad (R-Smad) phosphorylation. This is followed by translocation of R-Smads and the common mediator Smad (Co-Smad) complexes in the nucleus to regulate gene transcription (Moustakas et al., 2001). By contrast, inhibitory Smads (I-Smads) antagonize the activity of the R-Smads by preventing phosphorylation of R-Smads (Hill, 1999).

## TGF-β1-SMAD Pathway

Smads separate into different classes with regards to their functions: two TGF-β R-Smads (Smad2 and Smad3), three bone morphogenetic protein (BMP) R-Smads (Smad1, Smad5, and Smad8), one Co-Smad (Smad4) and two I-Smad (Smad6 and Smad7) (Hill, 1999; Heldin and Moustakas, 2012).

## R-SMADS

Smad2 and Smad3 are extensively studied in the TGF-β1 facilitated fibrotic response using various animal models and in human kidney disease, including diabetic (Isono et al., 2002; Fujimoto et al., 2003; Li et al., 2004; Chung et al., 2010; Chen et al., 2011) and obstructive nephropathy (Terada et al., 2002; Lan et al., 2003; Sato et al., 2003; Huang et al., 2008a; Chung et al., 2009), remnant kidney disease (Hou et al., 2005; Yang et al., 2010), hypertensive nephropathy (Wang et al., 2006), drug-associated nephropathy, and immunologically mediated glomerulonephritis (Ka et al., 2007; Huang et al., 2008b).

TGF-β1/Smad3 signaling mediates transcription of multiple downstream genes, such as the collagen chains ColIa1, ColIa2, ColIIIa1, ColaVa2, ColVIa1, and ColVIa3, and tissue inhibitor of metalloproteinases (TIMP)-1 (Verrecchia et al., 2001). The deletion of Smad3 in mice suppresses fibrosis in rodent models of kidney disease (Fujimoto et al., 2003; Sato et al., 2003; Zhou et al., 2010).

Relative to Smad3, the function of Smad2 in renal fibrosis is not fully elucidated. Because of the unavailability of Smad2 knock out (KO) mice, conditional kidney tubular epithelial cells Smad2 KO mice were generated by crossing the Smad2 floxed mouse with the kidney-specific promoter (Cadherin 16)-driven Cre transgenic mouse (Shao et al., 2002). Unexpectedly, deletion of Smad2 in tubular cells significantly enhances fibrosis, with an associated elevated Smad3 signaling in the UUO mouse model (Meng et al., 2010). Similarly, Smad2-/- fibroblasts have an increased fibrotic response (Meng et al., 2010). Additional evidence has shown that Smad3, but not Smad2, mediates fibrotic process (Wang et al., 2006; Yang et al., 2009, 2010; Chung et al., 2010; Zhou et al., 2010). Hence Smad2 and Smad3 may have distinct roles in mediating the fibrosis upon exposure to TGF-β1.

Among the R-Smads, BMP R-Smads (1, 5, 8) mediate the development of kidney and renal cell cancer (Oxburgh and Robertson, 2002; Blank et al., 2008; Markic et al., 2010). The BMP-7-Smad1/5/8 pathway has been shown to accelerate ECM deposition in the kidneys of unilateral ureteral obstruction (UUO) rats (Cao et al., 2015). The activin receptor-like kinase (ALK)-1/Smad1/5 pathway may influence ECM protein expression in several cell types, such as rat myoblasts, hepatocytes, and human chondrocytes (Munoz-Felix et al., 2013). However, the role of BMP R-Smads in fibrotic disorders remains largely unknown.

## CO-SMAD (SMAD4)

Smad4 promotes TGF-β1 signaling by dimerizing with R-Smads and facilitating nuclear translocation (Massague and Wotton, 2000; Gomez-Puerto et al., 2019). Deleting Smad4 from renal tubular cells alleviates renal fibrosis in a mouse model of UUO by suppressing Smad3 function (Meng et al., 2012). In mesangial cells, the loss of Smad4 inhibits TGF-β1 induced ECM accumulation (Tsuchida et al., 2003).

## I-SMADS

Smad 6 and Smad7 are inhibitory mediators in the TGF-β1 signaling pathway. They provide a negative feedback loop through multiple mechanisms, including competing with R-Smads in activating the receptors by associating directly with TGFβRI (Hanyu et al., 2001; Nakayama et al., 2001), indirectly affecting the activity of TGFβRI by cooperation with BMPs (Murakami et al., 2003; Yan et al., 2009), interference in the formation of R-Smad/Co-Smad complex (Hata et al., 1998; Yan et al., 2016) and abolishing transcription in the nucleus (Lin et al., 2003; Zhang et al., 2007).

The deletion of Smad7 accelerates fibrogenesis in a number of mouse models, including UUO (Chung et al., 2009), diabetic (Chen et al., 2011), and hypertensive nephropathy (Liu et al., 2013). However, the importance of Smad6 in renal fibrogenesis is unclear.

## Non-SMAD Pathways

TGF-β1 also independently and directly activates other pathways, such as Ras/Raf/extracellular-signal-regulated kinase (ERK)/ mitogen-activated protein kinase (MAPK) pathways, c-Jun N-terminal kinase (JNK), p38 MAPK signaling and Rho-like GTPase signaling pathways (Loeffler and Wolf, 2014).

TGF-β1 increases phosphorylation of tyrosine residues on TGFRs (I and II) and recruits ERK through Ras, Raf, and their downstream MAPK cascades. Specifically, ERK regulates target gene transcription through its downstream transcription factors in conjunction with Smads to control epithelial-mesenchymal transition (EMT) (Lee et al., 2007). ERK also regulates the activity of R-Smads, including Smad1, Smad2, and Smad3 (Kretzschmar et al., 1997, 1999; Funaba et al., 2002; Matsuura et al., 2005). Moreover, ERK is involved in the autoinduction of TGF-β1 via distinct transcriptional and translational mechanisms in tubular epithelial cells (Zhang et al., 2006). These studies suggest a dominant role of ERK in the non-Smad mediated transduction of TGF-β1.

The Rho-like GTPases, including RhoA, Rac, and Cdc42, play crucial roles in controlling dynamic cytoskeletal organization, cell motility, and gene expression through a variety of effectors (Jaffe and Hall, 2005). In addition to MAPK pathways, RhoA is a vital regulator, which can be activated by TGF-β1 via either Smad-dependent or independent pathways to promote stress fiber formation during EMT (Bhowmick et al., 2001a; Edlund et al., 2002).

JNK and p38 MAPK pathways are the best characterized non-Smad pathways involved in renal fibrosis. TGF-β1 can rapidly activate JNK and p38 MAPK via MAPK kinase (MKK) 4 and MKK 3/6, respectively (Frey and Mulder, 1997; Engel et al., 1999; Hanafusa et al., 1999; Hocevar et al., 1999; Sano et al., 1999; Bhowmick et al., 2001b; Yu et al., 2002). The activated JNK/p38 conjuncts with Smad2/3 to regulate apoptosis and EMT by controlling the activities of downstream transcription factors (Shibuya et al., 1998; Liao et al., 2001; Bakin et al., 2002; Yu et al., 2002; Edlund et al., 2003; Yamashita et al., 2008; Zhang, 2009). The phosphorylated JNK also regulates Smad 3 activity directly (Zhang, 2009; Grynberg et al., 2017).

## Receptor-Interacting Serine/Threonine-Protein Kinase (RIPK) 3

The RIP kinase family contains seven members, each of which possesses a homologous kinase domain. To date, the functions of RIPK4–7 are poorly understood (Zhang et al., 2010). RIPK2 is a mediator of mucosal immunity. Extensive studies have clarified the importance and physiological roles of RIPK1 and RIPK3 in inflammation and cell death (Christofferson et al., 2014; Newton, 2015).

The RIPK3 gene is located on chromosome 14 in both humans and mice (Kasof et al., 2000; Shlomovitz et al., 2017). This gene encodes a 518 amino acid (aa) protein with a molecular mass of 57 kDa in humans (Sun et al., 1999), whereas it encodes a 486 aa protein of 53 kDa in mice (Pazdernik et al., 1999). RIPK3 is a threonine/serine protein kinase that shares almost 30% identity and 60% with the other two RIPK members, RIPK1 and RIPK2 (Sun et al., 1999; Yu et al., 1999). Compared with RIPK2, RIPK3, and RIPK1 have a unique C-terminal RIP homotypic interaction motif (RHIM) (Sun et al., 1999), which enables homotypic protein interactions (Sun et al., 2002).

To date, several phosphorylation sites of RIPK3 have been identified. The human Ser227 site (Thr231/Ser232 for mouse RIPK3) and Ser199 site (Ser204 in mouse) are particularly crucial for the activation of its downstream substrate in the necroptosis pathway, mixed-lineage kinase domain-like (MLKL) (He et al., 2009; Sun et al., 2012; Chen et al., 2013; McQuade et al., 2013).

## RIPK3 in Necroptosis

In response to physiological signals and pathological stimuli, cell death is crucial to maintaining homeostasis. To date, several types of cell death have been identified. Among them, necrosis is a type of cell death characterized by loss of intracellular contents and the triggering of subsequent inflammatory response. For many years, necrosis was considered to be accidental and, therefore, unregulated cell death (Proskuryakov et al., 2003; Festjens et al., 2006; Zong and Thompson, 2006; Vandenabeele et al., 2010). The recognition that necroptosis is programmed necrosis that has provided new insights into necrosis-initiated cell death. Necroptosis is mediated by the dimerization of RIPK1-RIPK3, which forms the necrosome associated with the downstream expression of MLKL (Li et al., 2012; Newton and Manning, 2016; Weinlich et al., 2017). RIPK1 and RIPK3 both possess RHIM domains, with bilateral interaction between RIPK1 and RIPK3 (Li et al., 2012; Mompean et al., 2018). Subsequently, the necrosome facilitates the aggregation of phosphorylated RIPK3 and phosphorylation of MLKL by RIPK3 (Li et al., 2012; Newton and Manning, 2016; Weinlich et al., 2017). The phosphorylated MLKL translocates to the cell membrane and thus promotes necroptosis (Li et al., 2012).

In contrast to the obligatory involvement of RIPK3, RIPK1 is not always required to cause necroptosis. The M45-mutant strain of murine cytomegalovirus (MCMV) infection causes RIPK3 activation in the absence of activation of RIPK1 (Upton et al., 2010, 2012). There is also evidence that the RHIM-containing protein (ICP) 6 protein of herpes simplex virus 1 recruits RIPK3 directly and independent of RIPK1 (Wang et al., 2014b). In addition, RIPK1 may have dual influences on cell death by both promoting necroptosis and protecting cells from death under certain conditions (Filliol et al., 2017).

## RIPK3 in Kidney Fibrosis

To date, few studies have investigated the role of RIPK3 in kidney fibrosis, and most of them have not dissected the role of RIPK3 from necroptosis. The majority of these studies used acute injury models where there is known increased necroptosis.

The RIPK1 inhibitor necrostatin-1 reduces renal ischemia and reperfusion injury (Shen et al., 2019) and sepsis-associated acute kidney injury (Dong et al., 2018). Lacking RIPK3 protects kidney tubular injury in the sepsis-induced acute kidney injury (Sureshbabu et al., 2018). The deletion of either RIPK3 or MLKL prevents kidney damage in the oxalate crystal-induced AKI (Mulay et al., 2016) and kidney ischemia-reperfusion injury mouse models (Moerke et al., 2019). However, blockade of MLKL in folic acid-induced AKI (Martin-Sanchez et al., 2017) and 7-day unilateral ureteral obstruction (UUO) models (Imamura et al., 2018) failed to protect against kidney injury. Hence blockade of RIPK1, RIPK3, or MLKL may have differential benefits depending on the model under study.

Necrostatin-1 reduces interstitial fibrosis in a mouse model of UUO (Xiao et al., 2017) by inhibiting necroptosis, associated with lower protein and mRNA expression of RIPK, RIPK3, and MLKL and TGF-β1. In parallel, collagen accumulation and fibroblast activation (Xiao et al., 2017) were reduced. This study showed the integral relationship between necroptosis and TGF-β1 activation leading to renal fibrosis.

## RIPK3 in Apoptosis and Inflammation

Under certain conditions, RIPK3 also serves as a pro-apoptosis adaptor, which recruits RIPK1 and Fas-associated protein with death domain (FADD) to activate caspase 8 and thus apoptosis. This effect relies on the involvement of caspase 8 when RIPK3 is inactive, or MLKL is absent (Mandal et al., 2014; Newton et al., 2014). RIPK3 deficient animals develop normally, whereas mice expressing catalytically inactive RIPK3 ^D161N^ die around embryonic day 11.5 from increased RIPK1- and caspase-8-dependent apoptotic injury (Newton et al., 2014). Similar effects are observed in a study using compounds that selectively inhibit RIPK3 interaction with caspase 8 independent of pro-necrotic kinase activity and MLKL (Mandal et al., 2014).

Recent studies also identify that RIPK3 is an essential mediator in NOD-, LRR- and pyrin domain-containing protein (NLRP) 3 inflammasome formation (Wang et al., 2014a; Lawlor et al., 2015; Chen et al., 2018; Guo et al., 2019). In LPS-treated mouse bone marrow-derived dendritic cells, activation of the NLRP3 inflammasome was initiated by necroptosis (Kang et al., 2014). In a podocyte cell line, the RIPK3 specific inhibitor GSK’872 blocked both the necroptosis pathway and the NLRP3 inflammasome activation (Guo et al., 2019). These indicate the RIPK3 mediated NLRP3 inflammasome can be dependent of the necroptosis. Specifically, RIPK3–MLKL triggers NLRP3 activation when the activation of caspase 8 is reduced (Lawlor et al., 2015). In this setting, RIPK3 activity is required. RIPK3 can also promote NLRP3 inflammasome independent of the MLKL and RIPK3 kinase activity when caspase 8 is active (Lawlor et al., 2015). Collectively, RIPK3 mediated NLRP3 activation can be in both a necroptosis-independent and -dependent manner, depending on the levels of caspase-8 activity.

## RIPK3 and TGF-β1

**FIGURE 1:**
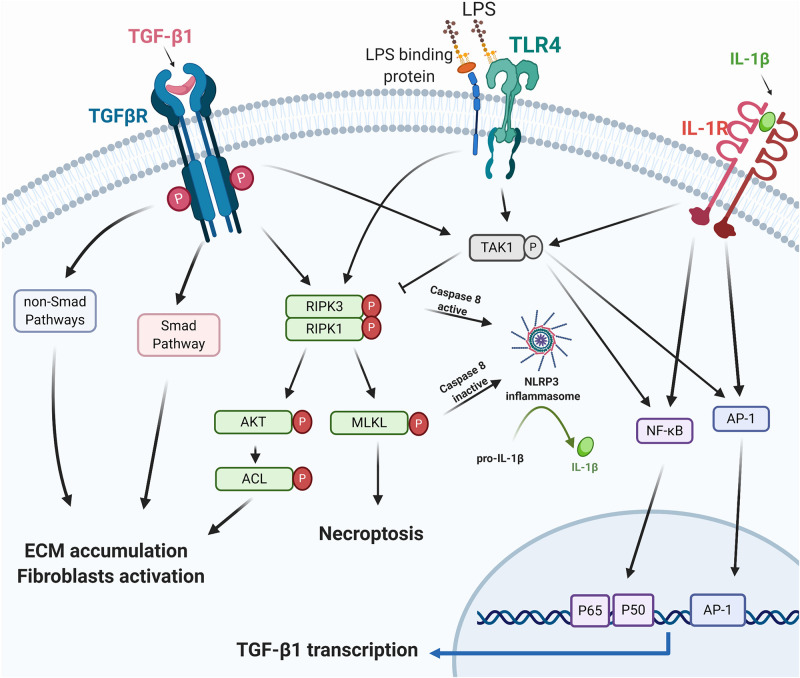
RIPK3 and TGF-β1. TGF-β1 exhibits its biological function via the canonical Smad/non-Smad pathways or TAK1/necrosome/AKT/ACL signaling to mediate ECM accumulation and fibroblast activation. Necroptosis or RIPK3 facilitates NLRP3 inflammasome assembly, triggers mature IL-1β secretion, and promotes the TGF-β1 transcription via the IL-1β regulated AP-1 and NFκB pathway (Lee et al., 2006). IL-1β, TGF-β, and TLR signaling pathways all activate TAK1 and its regulated inflammatory mediators (Kim and Choi, 2012; Fechtner et al., 2017). RIPK3, Receptor-interacting serine/threonine-protein kinase 3; TGF-β1, transforming growth factor beta-1; TAK1, TGF-β-activated kinase 1; AKT, protein kinase B; ACL, ATP citrate lyase; ECM, extracellular matrix; TLR4, toll-like receptor 4; LPS, lipopolysaccharides; NLRP3, NOD-, LRR- and pyrin domain-containing protein 3; IL-1β, interleukin-1β; AP-1, activator protein 1; NFκB, nuclear factor-kappa B. Created with BioRender.com.

Necrostatin-1 in a mouse UUO model attenuates IL-1β, TNF-α, and TGF-β1 levels (Xiao et al., 2017). In contrast, another study demonstrated that RIPK3 deficiency in the same UUO model prevents renal fibrosis without altering the mRNA expression of interleukin (IL)-1β, tumor necrosis factor (TNF)-α, and TGF-β1 (Imamura et al., 2018). These conflicting results may indicate that IL-1β, TNF-α, and TGF-β1 are “co-existing” as the downstream cytokines in the RIPK3 signaling. Mature IL-1β, the critical effector of the NLRP3 inflammasome (Jo et al., 2016; Kelley et al., 2019), has been demonstrated to increase TGF-β1 transcription (Lee et al., 2006). As described above, RIPK3 can regulate NLRP3 inflammasome (Lawlor et al., 2015). We, therefore, hypothesize that the “on/off switch” of RIPK3 in regulating TGF-β could be NLRP3 inflammasome activation (Figure 1). The trigger to promote NLRP3 inflammasome activation in RIPK3 signaling remains to be elucidated.

*In vitro* studies using NIH 3T3 fibroblasts, RIPK3 targeted siRNA, the RIPK3 inhibitor GSK’872 or necrostatin-1 abolished TGF-β dependent ECM and α- smooth muscle actin (α-SMA) expression (Imamura et al., 2018), suggesting that the necrosome RIPK1/RIPK3 is a downstream regulator of TGF-β in stimulating ECM deposition and fibroblast activation. The necrosome/RIPK3- Protein Kinase B (AKT)-dependent ATP citrate lyase (ACL) pathway has previously been identified as downstream of TGF-β (Imamura et al., 2018).

TGF-β-activated kinase 1 (TAK1), interacts with TGF-β1 and contributes to the development and progression of organ fibrosis through TGF-β1/TAK1/MKK3/p38MAPK, TGF-β1/TAK1/MKK4/JNK, and TGF-β1/TAK1/NFκB pathways (Kim and Choi, 2012; Biesemann et al., 2015; Li et al., 2017; Wu et al., 2017; Zhou et al., 2018; Bao et al., 2019). Few studies of TAK1 on necroptosis have been reported, and these mostly report on RIPK1-dependent cell death. A recent study explored TAK1 regulated endothelial necroptosis in tumor progression and showed that TAK1 deficiency increases necroptosis and RIPK3 expression in endothelial cells in both *in vitro* and *in vivo* studies (Yang et al., 2019). Endothelial knockout of RIPK3 or MLKL abolishes the effects of TAK1-deficiency on the enhancement of necroptosis, suggesting an inhibitory role of TAK1 on necroptosis (Yang et al., 2019). TAK1 may, therefore, negatively regulate the necroptosis in the TGF-β1 signaling network (Figure 1).

## Implications for Anti-Fibrotic Therapy

TGF-β1-specific, humanized, neutralizing monoclonal antibody added to RAAS inhibitors failed to slow the progression of diabetic nephropathy (Voelker et al., 2017). Therefore, targeting the full spectrum of downstream TGF- β1 signaling to prevent renal disease is unlikely to be fruitful, and the development of blockers of more targeted downstream pathways, such as the RIPK3/necroptotic pathway may be more beneficial.

To date, several small-molecule compounds (Li et al., 2014; Fauster et al., 2015; Martens et al., 2017, 2020; Park et al., 2018; Pan et al., 2019; Hart et al., 2020) have been developed to inhibit RIPK3 activity, providing an impressive toolbox for the investigation of the role of RIPK3 in diverse tissues. These inhibitors of RIPK3 can be divided into three types: ATP mimetic inhibitors targeting the active ATP-binding site in the kinases located between two catalytic domain lobes (type I), targeting the inactive states (type II), and unclassified inhibitors (Muller et al., 2015; Martens et al., 2020; Table 1).

**TABLE 1:** The RIPK3 inhibitors (Martens et al., 2020).

***Inhibitor types***	***Inhibitors***
*Type I*	Dabrafenib
	GSK’843
*Type II*	Sorafenib
	Ponatinib
	HS-1371
	GSK’067
	GSK’074
	Inhibitor 9
	Inhibitor 18
*Unclassified*	DCC-2036
	GSK’840
	GSK’872
	ZINC71828321
	ZINC72474191
	ZINC72454060
	GW’39B

GSK’872 is the most widely used cell-permeable inhibitor of the RIPK3-selective kinase, with >1,000-fold selectivity over a vast majority of more than 300 other kinases (Kaiser et al., 2013). GSK’872 binds the kinase domain and inhibits kinase activity with high specificity, targeting a broad range of pro-necrotic stimuli (Mandal et al., 2014) and has been used to specifically inhibit RIPK3 (Lu et al., 2017; Chen et al., 2018; Imamura et al., 2018).

The serine/threonine kinase B-Raf ^V600E^ inhibitor dabrafenib is the only type I RIPK3 inhibitor approved for clinical use (Rheault et al., 2013; Li et al., 2014; Sugaya et al., 2019). Previous studies have reported that dabrafenib is a selective RIPK3 inhibitor in various models, including human hepatocytes (Li et al., 2014), mouse models of acetaminophen-caused liver injury (Li et al., 2014), and ischemic brain injury (Cruz et al., 2018). In addition, dabrafenib is a well-known inhibitor of B-Raf, which suppresses the downstream Ras/Raf/ERK/MAPK pathway (Spagnolo et al., 2014), which has been approved for clinical use for the treatment of non-small cell lung cancer expressing B-Raf ^V600E^ mutations and in melanoma (Odogwu et al., 2018). Inhibition of Raf kinase has found to attenuate renal fibrosis (Xu et al., 2012b; Chen et al., 2019).

Collectively, inhibition of RIPK3 is a promising anti-fibrotic strategy. RIPK3 facilitates necrosome and necroptosis. RIPK3 stimulates downstream activation of TGF-β1 cascades and regulates TGF-β1 transcription through NLRP3 inflammasome activation. Given inhibitors of RIPK3 are already approved for use in patients with non-small cell lung cancer and melanoma, an accelerated route to market in patients with CKD should be available if early phase clinical studies prove positive.

## Author Contributions

YS wrote the manuscript. YS, CP, XC, and CH provided the critical discussion of the manuscript. YS and CP revised the manuscript.

## Conflict of Interest

The authors declare that the research was conducted in the absence of any commercial or financial relationships that could be construed as a potential conflict of interest.
