# Corrigendum: RIPK3: A New Player in Renal Fibrosis

**DOI:** 10.3389/fcell.2021.699073

**Published:** 2021-06-09

**Authors:** Ying Shi, Xinming Chen, Chunling Huang, Carol Pollock

**Affiliations:** ^1^Department of Nephrology, School of Medicine, Stanford University, Palo Alto, CA, United States; ^2^Kolling Institute of Medical Research, Sydney Medical School, The University of Sydney, Sydney, NSW, Australia

**Keywords:** RIPK3, receptor interacting serine/threonine-protein kinase 3, renal fibrosis, TGF-β1, necroptosis, dabrafenib

In the original article, there was a mistake in the legend for **Figure 1** as published. We neglected to include that **Figure 1** was created with BioRender.com. The correct legend appears below.

**Figure 1**. RIPK3 and TGF-β1. TGF-β1 exhibits its biological function via the canonical Smad/non-Smad pathways or TAK1/necrosome/AKT/ACL signaling to mediate ECM accumulation and fibroblast activation. Necroptosis or RIPK3 facilitates NLRP3 inflammasome assembly, triggers mature IL-1β secretion, and promotes the TGF-β1 transcription via the IL-1β regulated AP-1 and NFκB pathway (Lee et al., 2006). IL-1β, TGF-β, and TLR signaling pathways all activate TAK1 and its regulated inflammatory mediators (Kim and Choi, 2012; Fechtner et al., 2017). RIPK3, Receptor-interacting serine/threonine-protein kinase 3; TGF-β1, transforming growth factor beta-1; TAK1, TGF-β-activated kinase 1; AKT, protein kinase B; ACL, ATP citrate lyase; ECM, extracellular matrix; TLR4, toll-like receptor 4; LPS, lipopolysaccharides; NLRP3, NOD-, LRR- and pyrin domain-containing protein 3; IL-1β, interleukin-1β; AP-1, activator protein 1; NFκB, nuclear factor-kappa B. Created with BioRender.com.

The authors apologize for this error and state that this does not change the scientific conclusions of the article in any way. The original article has been updated.

